# Exclusive and adjuvant radiotherapy in breast cancer patients with synchronous metastases

**DOI:** 10.1186/1471-2407-10-630

**Published:** 2010-11-17

**Authors:** Romuald Le Scodan, David Ali, Denise Stevens

**Affiliations:** 1 Department of Radiation Oncology, Institut Curie Hôpital René Huguenin, Saint Cloud, France; 2 Medical Statistics, Institut Curie Hôpital René Huguenin, Saint Cloud, France

## Abstract

**Background:**

Data from the Surveillance, Epidemiology, and End Results program and the European Concerted Action on survival and Care of Cancer Patients (EUROCARE) project indicate that about 6% of women newly diagnosed with breast cancer have stage IV disease, representing about 12 600 new cases per year in the United States in 2005. Historically, local therapy of the primary tumor in this setting has been aimed solely at symptom palliation. However, several studies suggest that surgical excision of the primary tumor can prolong these patients' survival.

**Discussion:**

Exclusive locoregional radiotherapy is an alternative form of locoregional treatment in this setting and may represent an effective alternative to surgery in this setting. Here we discuss current issues regarding exclusive and adjuvant locoregional radiotherapy in breast cancer patients with synchronous metastases.

**Summary:**

Several studies suggest that surgery or exclusive irradiation of the primary tumor is associated with better survival in breast cancer patients with synchronous metastases and that exclusive locoregional radiotherapy may represent an effective alternative to surgery in this setting. Results of well-designed prospective studies are needed to re-evaluate treatment of the primary breast tumor in patients with metastases at diagnosis, and to identify those patients who are most likely to benefit.

## Background

Data from the Surveillance, Epidemiology, and End Results program and the European Concerted Action on survival and Care of Cancer Patients (EUROCARE) project indicate that about 6% of women newly diagnosed with breast cancer have stage IV disease, representing about 12 600 new cases per year in the United States in 2005 [1,2]. The 5-year overall survival rate among such patients rarely exceeds 20% [3]. Survival can be improved by endocrine therapy, chemotherapy, and biological therapy [4,5]. Local treatment is often recommended to prevent or relieve symptoms but is traditionally considered to have no noteworthy impact on survival [4,5]. However, several recent observational studies have shown that 35% to 60% of breast cancer patients with stage IV disease at diagnosis receive treatment for the primary tumor, consisting mainly of surgery [6-19]. The results of these studies, coming from the SEER database, the National Cancer Database (NCDB), the Geneva Tumor Registry and several large comprehensive cancer center databases, show that surgery of the primary tumor was associated in most series with a relatively constant reduction in the risk of death of about 40% [6-13,15-19] (table 1).

**Table 1:** Retrospective studies evaluating the treatment of the primary tumor in breast cancer patients with synchronous metastases

Authors (ref)	Database	Years of inclusion	Local treatment	Number of patients total/local treatment/no local treatment	Characteristics associated with a higher OS rate in multivariate analysis	Multivariate Analysis of Overall Survival Hazards Ratio for Death (95%CI) with local treatment
Kahn et al. [13]	National cancer database of the American college of surgeons	1990-1993	S	16023/9162/6861	S, systemic therapy, number of metastatic sites	HR OS (RO) = 0.61 (0.58-0.65) HR (R1) = 0.751 (0.71-0.793)

Gnerlich et al. [11]	SEER	1988-2003	S	9734/4578/5156		HR OS = 0.57 (0.55-0.60)

Bafford et al. [7]	Dana Farber Cancer Institute, Brigham and Women's Hospital and Massachusetts General Hospital	1998-2005	S	147/61/86	S, ER+, Her2+, no CNS metastasis	HR OS = 0.47 (p = 0.003)

Shien et al. [18]	National Cancer Center Hospital	1962-2007	S	326/160/184	S, age <50, soft tissue or bone metastasis	HR OS = 0.89 (0.79-1)

Blanchard et al. [8]	Laboratory of the university of texas health science center	1973-1991	S	395/242/153	S, ER +, PR+, number of metastatic sites	HR OS = 0.609 (0.489-0.757)

Fields et al. [10]	Washington university medical center	1996-2005	S	409/287/222	S, Bone only metastasis	HR OS = 0.53 (0.42-0.67) No difference in time to metastatic progression between the 2 groups

Babiera et al. [6]	MD Anderson cancer center	1997-2002	S	224/82/142	Only one site of metastasis, HER2+, Caucasian ethnicity	HR OS = 0.5 (0.21-1.19) HR TTFP = 0.54 (0.38-0.77, p = 0.0007)

Hazard et al. [12]	Lynn Sage Breast Center (Northwestern memorial Hospital)	1995-2005	S	111/47/64	NA	HR OS = 0.798 (p = 0.52) HR TTFP = 0.49 (p = 0.015)

Cady et al. [9]	Massachusetts General Hospital and Birgham and Women's Hospital	1970-2002	S	622/234/388	Young age, RH +, bone only metastasis	Matched-pair analysis: benefit of surgery p < 0.0001

Ruiterkamp et al. [17]	Eindhoven Cancer Registry	1993-2004	S	728/288/440	Surgery, age, no more than one metastatic site, no concomitant disease(p = 0.06), systemic therapy	HR OS = 0.62 (95%CI = 0.51-0.76)

Leung et al. [19]	Medical College of Virginia Campus of Virginia Commonwealth University,	1990-2000	S	157/52/105	Chemotherapy	No benefit in multivariate analysis

Rapiti et al. [16]	Geneva cancer registry	1977-1996	S	300/127/173	Age < 60, none N3, ER+, none visceral metastasis, none CNS metastasis, hormonal treatment, surgery with negative margins	HR OS = 0.6 if R0 (0.4-1.0) NS if R1

Le Scodan et al. [14]	René Huguenin Cancer Center	1984-2004	RT	581/320/261	Only one metastatic site, young age, LRT, no visceral metastases, N0	HR OS = 0.7 (0.58-0.85)

S: surgery of the primary tumor, RT: radiation therapy, LRT: locoregional treatment, HR: hazard ratio, OS: overall survival, TTFP: time to first progression, 95%CI: 95% confidence interval, MST: median survival time, CNS: central nervous system, ER+: tumor positive estrogen receptor, PR+: tumor positive progesteron receptor, Her2+: Her2 positive status, R0: surgery with negative margins, R1: surgery with positive margins, N0: clinical N0 lymph node status

Locoregional treatment may also consist of exclusive locoregional radiotherapy with the added advantage of being a conservative treatment. Two recent studies have evaluated the impact of locoregional radiotherapy directed to the breast and regional lymphatics among breast cancer patients with synchronous metastases [14,20]. In contrast, the role of postoperative radiotherapy in this setting is poorly documented. The main objective of this review is to highlight current issues regarding exclusive and adjuvant locoregional radiotherapy in breast cancer patients with synchronous metastases.

## Discussion

### Locoregional treatment in metastatic cancer and pathophysiological hypotheses

Resection of the primary tumor has been linked to better survival in several metastatic malignancies. Two phase III randomised controlled trials comparing medical treatment alone versus medical treatment plus nephrectomy for metastatic renal carcinoma showed a significant overall survival benefit among patients whose primary tumor was removed [21,22]. Excision of the primary tumor is also known to be beneficial in stomach cancer [23] melanoma [24] colon cancer [25,26], and ovarian cancer [27]. Similarly, several recent observational studies have shown a survival advantage among breast cancer patients with stage IV disease at diagnosis whose primary tumor was completely excised [6-19]. The largest series was published by Khan et al., who investigated the use and impact of local therapy among 16 023 breast cancer patients with synchronous metastases registered in the National Cancer Data Base of the American College of Surgeons between 1990 and 1993 [13]. Complete surgery of the primary tumor, i.e. with free margins, was associated with a 39% reduction in the risk of death: the 3-year survival rate was 35%, compared to 26% and 17.3%, respectively, among patients with positive margins and patients who did not receive surgery (p < .0001). This survival benefit of breast surgery persisted in multivariate analysis. Similar conclusions were reached by Rapiti and coworkers: among 300 women included in the Geneva Cancer Registry between 1977 and 1996, complete surgical resection of the primary tumor significantly improved overall survival [16]. Analysis of the 1988-2003 SEER dataset [11] and smaller series from other institutional databases, such as the Baylor College [8] and MD Anderson [6] also point to a benefit of surgery for stage IV breast cancer (table1). Several mechanisms potentially support the use of local treatment in the metastatic setting.

First, removal of the primary tumor may reduce the total tumor burden, increasing the effectiveness of chemotherapy, and limit an additional reseeding of tumor if one considers that the primary tumor is the only continuous source of metastases and that systemic spread from metastatic lesions is less likely [28]. Total tumor burden plays a central role in survival, since the number of metastatic sites and the number of metastasis at a given site is strongly correlated with survival of breast cancer patients [29-32]. There is also a correlation between the level of circulating tumor cells and the prognosis of metastatic breast cancer [33,34]. Furthermore, it has been reported that chromosomal abormalities in circulating tumor cells isolated from patients with metastatic epithelial cancers match those in the primary tumor, indicating that circulating cells are derived from the primary tumor [28]. Second, removal of the primary tumor may make metastases more chemosensitive, by inducing an angiogenic surge (thereby increasing tumor vascularisation and drug penetration), by removing necrotic tissue and non vascularised tumor cells (which are classically less sensitive to chemotherapy and radiation therapy) and by eliminating breast cancer stem cells from the primary tumor, limiting the emergence of chemoresistant cell lines [29-31,35]. Third, removal of the primary helps to restore immunity and to improve nutrional status. Indeed, some tumors, including breast cancer, can induce an immunosuppressive state and influence metastatic disease progression possibly owing to cytokine secretion by tumor cells [35]. In a murine model, Danna et al. demonstrated that a primary tumor may influence metastatic disease progression through the release of immunosuppressive factors and that removal of the primary tumor may result in restoration of an immune response, even in the presence of metastatic disease [36]. Fourth, surgery or exclusive locoregional radiotherapy of the primary breast tumor effectively prevents uncontrolled chest wall and in-breast disease. Prospective randomized trials of postmastectomy radiotherapy have shown that local therapy in the form of chest wall and lymph node irradiation prolongs survival in node-positive non-metastatic women receiving tamoxifen or chemotherapy [37-39]. This suggests that local therapy impacts survival in breast cancer that is likely to be systemic and that uncontrolled local disease may act as a source of tumor reseeding, diminishing the effectiveness of systemic therapy. This is supported by the finding that the increased local recurrence rate after lumpectomy without radiotherapy translates into poorer 15-year survival [40]. Moreover, a randomized controlled trial showed that local recurence is predictive of distant dissemination [41]. In the study by Hazard, surgery strongly protected against uncontrolled chest wall disease, suggesting that the impact of local therapy on survival may be mediated by better local control [12].

Thus, both mechanisms -- a reduction in tumor burden by removing the primary tumor that serves as the source of tumor cell seeding, and better local control - may be involved. Indeed, these mechanisms are linked, because uncontrolled local disease may serve as a source of systemic tumor reseeding. Opposite to the proposed biological mechanisms in favor of treatment of the primary tumor, other theories have been proposed regarding the effect of surgical removal of the primary tumor on the growth kinetics of micrometastases. In contrast, several authors suggested that surgical resection of the primary breast tumor may accelerate relapse due either to removal of inhibitors of angiogenesis and/or the release of growth and immunosuppresive factors in response to surgical wounding [42-45]. However, the literature review of the retrospective studies evaluating the impact of surgical resection of the primary breast tumor does not support this point of view.

### Exclusive locoregional radiotherapy for the primary breast tumor

Exclusive locoregional radiotherapy (LRR) is an alternative form of locoregional treatment (LRT) in this setting, and has the advantage of breast conservation.

Several series support the use of LRR alone in the management of breast c ancer and have shown good locoregional control with local control rates of about 80% at 5 years [46-55]. For example, Dubray et al reported local control rates of 86% and 74% at 5 and 10 years respectively, in 398 (33 T1, 309 T2, 56 T3) breast cancer patients treated conservatively at Hôpital Henri Mondor (France) by an initial course of external irradiation (45 Gy, 25 fractions, 5 weeks) followed by interstitial iridium-192 implant for a further 37 Gy to the tumor [46]. We recently studied the impact of LRT, consisting mainly of exclusive LRR, on the survival of breast cancer patients with synchronous metastases treated between January 1984 and December 2004 at Rene Huguenin Cancer Center, Saint Cloud, France [14]. Among 581 patients, 320 received LRT and 261 received no LRT. LRT consisted of exclusive LRR in 249 cases (78%), surgery of the primary tumor with adjuvant LRR in 41 cases (13%), and surgery alone in 30 cases (9%). Exclusive LRR delivered mean doses of 48.67 Gy (range:5-50) and 48.01 Gy (range: 5-50), respectively, to the affected breast and axillary and supraclavicular lymph nodes. Eighteen patients received hypofractionated LRR. One hundred fifty-eight patients (63.5%) received an additional dose to the primary tumor (mean dose: 22.95 Gy; range: 9-40; brachytherapy: 58 patients; external irradiation: 100 patients). One hundred twenty-four patients (42.7%) received an axillary boost (mean dose 16.7 Gy, range: 9-25) and 57 patients (20%) received a boost to the supraclavicular fossa (mean dose 11.17 Gy; range: 5-18). With a median follow-up of 39 months, the 3-year OS rates were 43.4% and 26.7%, respectively, among patients who received or not LRT (p = 0.00002). LRT was an independent factor of favourable outcome in multivariate analysis, taking into account the main cancer-related prognostic factors (hazard ratio, 0.70 [95%CI 0.58-0.85]; p = 0.0002). We also evaluated the adjusted HR for the effect of LRT from time 0 to 1 year and to 1 year or more. A similar beneficial association between LRT and a lower risk of death was observed, with an HR for death of 0.45 (95% CI, 0.32 to 0.65; *P *<.0001) from diagnosis to 1 year and an HR for death of 0.76 (95% CI, 0.61 to 0.96; *P *= .02) 1 year or more after diagnosis, suggesting that LRT clearly impacts OS and that the effect of treatment is not only a result of a treatment assignment bias. Althought few patients were treated with surgery alone, the median survival times and 3-year overall survival rates were 26 months and 46% (95% CI, 29.60% to 63.60%) among the 30 patients treated with surgery alone and 31 months and 41.5% (95% CI, 35.50% to 47.90%) among the 249 patients treated with exclusive LRR, suggesting that exclusive locoregional radiotherapy might be an effective treatment of the primary tumor.

Bourgier and colleagues from the Institut Gustave Roussy, Villejuif, France, recently reported the outcome of 239 breast cancer patients with synchronous metastases who received locoregional treatment of the primary tumor [20]. Two-thirds of the patients had a sole metastatic sites and 49% had non-visceral metastases at diagnosis. They evaluated the effect of local treatments (LRR alone: group 1; n = 147 versus surgery of the primary tumor with or without adjuvant radiotherapy: group 2; n = 92) on local control, overall (OS) and metastasis progression-free (MPFS) survival. Breast and regional lymphatics were irradiated daily with hypofractionation (30 Gy/10 fractions; 63% of Group 1 patients) or with conventional fractionation (50 GY/25 fractions; 32% of Group 2 patients) and a tumor boost was delivered to more than half of the patients. With a median follow-up of 6.5 years, the 3-year MPFS rates were 20% in group 1 and 39% in group 2; the 3-year OS rates were 39% and 57% respectively. However, no significant differences in MPFS or OS were observed between the two groups when adjusted on known prognostic factors. This study confirms that exclusive LRR is an effective alternative to surgery in the management of breast cancer patients with synchronous metastases.

### Role of adjuvant radiotherapy

In Kahn's series, radiation therapy was received by 5806 of the 16 023 women, but no information was provided on whether it was directed to the breast, the chest wall, or osseous or symptomatic metastatic sites [13]; Rapiti and colleagues reported that women who had surgery (and especially breast-conserving surgery) were more likely to have local radiotherapy too (21% vs 5%; P < 0.0001) [16]. Radiation therapy, delivered to 266 patients (89%), was associated with significantly improved survival in the multiadjusted model (hazard ratio for death without radiation therapy, 1.6 (95%CI: 1.0-2.5)), but the authors did not state whether irradiation was delivered to the breast and regional lymphatics or to treat metastatic sites. Gnerlich found that 41% of patients received radiation therapy in the surgery group, compared to 34% of patients in the no-surgery group, and that irradiation was associated with a reduction in the risk of death in univariate analysis (HR = 0.83, 95CI: 0.79-0.87), but it was unclear whether or not irradiation was a prognostic factor in multivariate analysis [11]. In the recent series reported by Ruiterkamp and colleagues, locoregional radiotherapy was not associated with better overall survival in multivariate analysis [17]. In the study by Le Scodan et al., the median survival times and 3-year OS rates were 26 months and 46% (95% CI, 29.60% to 63.60%) for the 30 patients treated with surgery alone, 31 months and 41.5% (95% CI, 35.50% to 47.90%) for the 249 patients treated with exclusive locoregional radiotherapy, and 39 months and 52.6% (95% CI, 37.60% to 67.20%) for the 41 patients treated with surgery followed by locoregional radiotherapy, respectively (*P *= 0.07) [14]. However, comparisons between a multimodality treatment targeting the primary tumor and regional lymphatics and surgery alone must be undertaken with care, owing to the potential selection bias. Thus, the possible benefit of post-operative radiotherapy is unclear. Several randomized trials have supported the use of hypofractionned whole breast radiotherapy and have shown good results in breast cancer patients with non-metastatic, node-negative disease [56,57]. If locoregional radiotherapy following surgery of the primary tumor is considered to be of significant interest in this metastatic setting, accelerated radiotherapy may represent an active alternative to normofractionnated schedules.

### Potential selection bias and ongoing phase III studies

The results of these recently published observationnal studies raises two possibilities: either local treatment of the primary provides a substantial survival benefit in women with metastatic breast cancer at diagnosis, or there is a strong and consistent selection bias driving the use of this treatment in women who have already factors of better outcome. All retrospective studies are likely to suffer from selection biases. Current clinical decision-making seems to reliably identify women who will do better, as most of the studies evaluating the impact of the treatment of the primary breast tumor in this context of metastatic disease showed an association between surgery or exclusive radiotherapy and known factors of good prognosis [6-19]. Indeed, women in the surgical groups were younger, had smaller tumors and fewer metastatic sites, and were more likely to have bone/soft tissue metastases rather than visceral disease. Finally, it is also possible that local treatment is a surrogate marker of more aggressive therapy overall, including more aggressive systemic therapy, translating into better survival. This possibility is supported by the fact that, in several studies, patients were more likely to receive radiotherapy [7,12,16,17] or chemotherapy [6,14,17] when they had treatment of the primary tumor. Thus, only a large prospective randomized trial could settle this issue. Given the relatively minor adverse effects associated with the treatment of the primary tumor, along with the relatively consistent survival benefit observed in the different retrospective studies of local surgery or radiation -- and the estimated/12,000 breast cancer patients with synchronous metastases diagnosed each year in the United States -- a prospective randomized trial is more than justified, although issues of design, feasibility and priority of such a trial are more complex. Such a study is under discussion among US and European cooperative groups and two randomized trials, one sponsored by the Turkish Federation of the National Societies for Breast Diseases and the other by Tata Memorial Hospital, India, are currently recruiting. The Turkish trial is intended to enroll 271 patients in a comparison of upfront surgery (mastectomy or breast conserving surgery with level I-II axillary clearance in clinically or sentinel lymph node positive patients) with adjuvant therapies and systemic therapy only [58,59]. In the systemic chemotherapy group, patients will only receive surgery to control local complications.The primary end-point is mortality and the secondary end point is the assessment of quality of life within the two groups. The estimated completion of this study is October 2012. The Tata Memorial Hospital trial should enroll 350 patients in a comparison of locoregional therapy (i.e. surgery and adjuvant radiotherapy if indicated) and no locoregional therapy, given after six cycles of anthracycline-based chemotherapy [60]. Primary endpoints are time to progression and overall survival, secondary are correlative science points such as change in angiogenics factors. The estimated study completion date is february 2011. In a preliminary report of this trial (NCT00193778) (125 patients: 53 pts randomized to surgery and 72 pts randomized to observation; median follow-up: 18 months), surgery of the primary tumor was not associated with better PFS or OS [61]. However, if positive, the results of these studies will be of interest not only to women with initial stage IV disease but also those with synchronous local and distant recurrences of previously treated breast cancer.

## Summary

Several studies suggest that surgery of the primary tumor is associated with better survival in breast cancer patients with synchronous metastases and that exclusive locoregional radiotherapy may represent an effective alternative to surgery in this setting. Results of well-designed prospective studies are needed to re-evaluate treatment of the primary breast tumor in patients with metastases at diagnosis, and to identify those patients who are most likely to benefit.

## Competing interests

The authors declare that they have no competing interests.

## Authors' contributions

RLS and DA performed data interpretation and wrote the manuscript. RLS, DA and DS approved the final manuscript.

## Pre-publication history

The pre-publication history for this paper can be accessed here:

http://www.biomedcentral.com/1471-2407/10/630/prepub
